# Online Consumer Surveys as a Methodology for Assessing the Quality of the United States Health Care System

**DOI:** 10.2196/jmir.6.1.e2

**Published:** 2004-01-20

**Authors:** Christina Bethell, John Fiorillo, David Lansky, Michael Hendryx, James Knickman

**Affiliations:** ^1^Kaiser Permanente Center for Health ResearchPortland ORUSA; ^2^(formerly) The Robert Wood Johnson Foundation, Princeton, NJ, USA; (currently) The Health Strategy Group IncChatham NYUSA; ^3^FACCT—Foundation for AccountabilityPortland ORUSA; ^4^Washington State UniversitySpokane WAUSA; ^5^The Robert Wood Johnson FoundationPrinceton NJUSA

**Keywords:** Quality of health care, data collection, Internet, health care surveys, consumers

## Abstract

**Background:**

Interest in monitoring the quality of health care in the United States has increased in recent years. However, the policy objectives associated with collecting this information are constrained by the limited availability of timely and relevant data at a reasonable cost. Online data-collection technologies hold the promise of gathering data directly and inexpensively from large, representative samples of patients and consumers. These new information technologies also permit efficient, real-time assessment in such areas as health status, access to care, and other aspects of the care experience that impact health outcomes.

**Objective:**

This study investigates the feasibility, validity, and generalizability of consumer online surveys to measure key aspects of health care quality in the United States.

**Methods:**

Surveys about the health and health care experiences of a general adult population and of adults with diabetes were administered online and by telephone. The online survey drew from a sample frame of nearly 1 million consumers and used a single e-mail notification. The random-digit-dial methodology included 6 follow-up calls. Results from the online sample were compared to the telephone sample and to national benchmark data.

**Results:**

Survey responses about quality of care collected using online and telephone methods were commensurate once they were weighted to represent the demographic distribution of the 2000 United States Census. Expected variations in health and health care quality across demographic and socioeconomic groups were largely observed, as were hypothesized associations among quality indicators and other variables. Fewer individuals were required to be contacted to achieve target sample sizes using online versus telephone methods. Neither method yielded representative cohorts of nonwhite individuals.

**Conclusions:**

Conclusions about the level and variations in health care quality in the United States are similar using data collected in this study compared to data collected using other telephone-based survey methods. As is typical for national telephone surveys conducted by the National Center for Health Statistics, stratified sampling and weighting of survey responses is necessary for results to be generalizable. Online methods are more appropriate for understanding health care quality than for conducting epidemiologic assessments of health in the United States.

## Introduction

Recent years have seen a marked increase of interest in monitoring the quality of health care in the United States. Congress has mandated the annual release of a National Healthcare Quality Report, which will include results from consumer-reported surveys on health care quality [1]. Congress, a presidential commission, and the National Quality Forum have all called for publication of consumer-centered quality performance information, and the administrator of the Medicare program has indicated the government's intention of releasing performance data for nursing homes, hospitals, and perhaps even physicians [2,3]. State Medicaid and State Children's Health Insurance Programs (SCHIP) are required to assess and report on quality of care provided to consumers enrolled in these programs [4,5].

### Need for Timely and Efficient Collection of Quality Information

These policy objectives are constrained by the limited availability of timely and relevant data at a reasonable cost. Often, information strategies for health care quality must rely on datasets defined and populated for other reasons, such as documentation of financial transactions, public health surveillance, or contractual oversight and audit. Seldom do such assessment systems address the health care quality concerns of patients and consumers, and rarely do they capture their experiences or attitudes.

Consequently, a tension exists between the policy objective of evaluating our success at creating a more-responsive health system that achieves priority health goals and our dependence on an information infrastructure unable to capture the necessary data to determine whether these goals are being achieved. Two trends offer some hope of resolving this tension. First, scientists have developed and validated an extensive library of patient survey instruments over the past 20 years. Tools now permit us to measure the performance of the health system along the dimensions of health care outcomes and the provision of clinically-appropriate, patient-centered care [6- 12]. Second, new information technologies hold the promise of gathering data directly and inexpensively from large, representative samples of patients and consumers. Online data-collection technologies also permit efficient, real-time assessment in such areas as health status, access to care, and other aspects of the care experience that affect health outcomes [13- 14]. Given the potential efficiencies and expediency of collecting data online, as well as growing limitations in telephone-based and/or mail-based surveys, it is clearly worthwhile—perhaps vital—that we develop and test online methods for capturing consumer-reported information on quality of health care.

### The Challenges of Web-Based Patient Surveys

All modes of consumer-survey administration entail challenges of measurement error, nonresponse error, and, particularly, coverage error. Online methods may be helpful in reducing some of these sources of error, but may also encounter new challenges in other sources of error.

#### Measurement Error

Web-based surveys introduce a new mode of interaction with respondents. The online experience involves both technical and contextual changes that may cause variations from how the same individuals would answer questions if presented in person, on the telephone, or by mail. Among technical differences are the presentation of questions and responses on computer screens, and variations in browser layouts, colors, text, and communication speeds. Contextual factors include users' ability to review and change prior answers, look ahead to other content, "multi-task," or start and stop during a session. Studies evaluating Web-based survey-mode effects have generally shown them to more closely resemble self-administered mail surveys than interviewer-administered telephone surveys, though with lower item nonresponse [13,15] and the potential for immediate data analysis and feedback to sponsors and respondents [16,17]. To ensure consistent user experiences and reduce measurement error, a consensus set of procedural recommendations analogous to those for mailed and telephone administered surveys is emerging for conducting Web-based surveys [18- 20].

#### Non-Response Error

Continuing changes in consumer telephone behavior have increased and redefined nonresponse error in phone surveys [21,22]. The common use of answering machines and of technologies for caller identification and unknown-caller blocking all contribute to nonresponse bias for telephone surveys. Consumer resistance to receiving telephone calls by telemarketers is reflected in the Do-Not-Call registry recently required by Congress and implemented by the Federal Trade Commission [23]. While survey researchers conducting surveys for not-for-profit or public-interest purposes are not prevented from calling individuals in this registry, the overarching resistance and resentment expressed by consumers regarding calls made to their home during evenings and weekends could generalize to a resistance to respond to calls to conduct these types of surveys.

Although researchers have begun to study the extent and causes of nonresponse error to e-mail and Web-based surveys, it is not well-documented and remains an especially-serious concern of methodologists when considering the use of the Web to conduct population-based surveys intended for use in policy contexts [21,23,24]. Documented reasons for nonresponse range from traditional questions of content interest to respondents' use of multiple e-mail accounts and defunct or infrequently-accessed e-mail accounts [21,25,26]. The emerging consensus procedures focus on the importance of repeat contacts, tracking nondelivery to e-mail accounts, and incentives to maximize response rates.

#### Incomplete Coverage

Errors are introduced in national telephone surveys because of households without telephones. Similarly, incomplete Internet access, or coverage, introduces error in national estimates based on online survey data. While the majority of Americans have access to the Web, there remain significant economic, cultural, and educational disparities. As of mid-2000, the US (United States) government estimated that only13% of persons with annual incomes under $15000 had Internet access in their homes, compared with 78% of those with incomes over $75000 [27]. Overall, whites enjoy greater Web access than do African-Americans and Hispanics [27]. These disparities may be rapidly changing, however. According to a 2001 UCLA report, 72% of all Americans have Internet access, including 65% with less than a high school education [28]. Certain demographics can be specifically targeted through Web technology—for example, customers of America Online, users of eHealth services, or populations sharing an e-mail domain who can be sent a request to complete a survey. Examples could be university-affiliated populations or employees of large companies. Also, larger proportions of low-income and elderly Americans increasingly use the Internet and can be sampled in online surveys [27].

Researchers advocate various ways of responding to the noncoverage and nonresponse challenges inherent in all survey administration modes, including Web-based surveys:

1. adjusting nonrepresentative completed samples according to characteristics known of both the starting sample and underlying populations [29- 30]
2. coupling participant recruitment using random-digit dialing with the efficiencies and interactivity of the Web [31]
3. restricting use of online data collection to studies of fully-covered populations (eg, university surveys and subscribers to specific Web sites)
4. delaying use of Web surveys until coverage improves and methodological developments take place [32].

The Robert Wood Johnson Foundation (RWJF) and the Foundation for Accountability (FACCT) recognized the potential value of conducting Internet-based surveys to calculate national estimates of health care quality. In 2000 and 2001, as part of the Robert Wood Johnson Foundation National Strategic Indicators Project (NSIP), online surveys were fielded to assess health care quality for children, teens, and adults with and without chronic conditions. Survey topics were identified as relevant for each of the 5 Foundation for Accountability Consumer Information Framework (CIF) domains (the basics, staying healthy, getting better, living with illness, and changing needs) and types of measures (health outcomes, appropriate clinical care, and patient-centered experience of care) [33]. The Consumer Information Framework has been adapted for use in the Congressionally-mandated National Health Care Quality Report [1] to structure the identification and communication of quality information to the public.

In this paper, we report the extent to which data derived from a national online sample of the general adult population and from a sample of adults with diabetes meet initial criteria for use in characterizing the performance of health care systems.

Four research questions are preliminary to the overall feasibility and validity of using Web-based surveys to estimate health care quality:

- Are online survey response rates (derived from a sampling frame recruited using opt-in Internet methods) of sufficient size and representation to estimate health care indicators for the US population?
- Do estimates of key health status and health care system quality variables demonstrate face validity, compared to other national studies?
- Do these estimates demonstrate concurrent validity, such that demographic and other correlates of health status and health care system performance match those observed in national telephone-based surveys?
- Are survey scale data collected online psychometrically reliable?

## Methods

This study reports both online and telephone-administered health care survey results for adults age 18 and over, as well as for adults with diabetes. Also used are data from adult respondents to the 1999 Behavioral Risk Factor Surveillance Survey (BRFSS) and the 1998 National Health Interview Survey (NHIS) administered by the US Centers for Disease Control and Prevention (CDC).

### Data Collection

#### Online Surveys

A market research firm, Common Knowledge, Inc, recruited a panel of approximately 1 million individuals, using Internet advertisements intended to attract a group with diverse demographic and psychographic characteristics. Approximately 70% of the panel was recruited online, the remaining 30% through traditional direct-mail and telephone contact. Panelists were invited to participate in only one study per month to prevent "professional" survey takers from responding and to minimize respondent fatigue.

Two waves of sampling and data collection took place for the general-adult and adult-diabetes online surveys. In the first wave, separate stratified random samples were drawn, each representing the US population along the dimensions of age, sex, and education using 4 age groups (18-24, 25-44, 45-64, over 65) and 4 educational groups (less than high school, high school/GED [General Equivalency Diploma], some college, college or more). A standard self-reported screening tool was used to identify individuals age 18 and over and those with diabetes. For the general adult survey, 13400 invitations were sent in the first wave of data collection. Diabetes-qualified respondents were screened as part of a larger effort to identify several chronic illnesses. Once a person qualified for one condition they were routed to complete the survey for persons with that condition until target sample sizes for each condition were achieved. As such, no sample-wide qualification rate for adult diabetes is available. A second wave of 1400 invitations oversampled individuals with Spanish surnames or who lived in zip-code areas with disproportionate numbers of African-Americans and/or Hispanics. An online survey research firm, E-valuations, Inc, sent invitations and collected data for both waves, using the sampling design and surveys developed by the Foundation for Accountability and the Robert Wood Johnson Foundation. Each respondent was given a unique 5-digit access code to ensure that the survey was taken only once. Those who completed it were entered into a drawing for a $250 cash prize. No reminder e-mails were sent, nor were nonworking or dormant e-mail addresses tracked.

#### Telephone Surveys

Adults age 18 and over constituted the sampling frame for the 2 telephone surveys. Wirthlin Associates, Inc identified individuals by means of traditional random telephone-survey methods, and used the sampling design and surveys developed by the Foundation for Accountability and the Robert Wood Johnson Foundation to conduct the surveys. Candidate telephone numbers were randomly selected and call attempts made until the target completed sample sizes of 400 for each survey was reached.

### Measures

This study evaluates the Internet methodology for both the general-adult and adult-diabetes samples, using demographic variables and the following topics. Sources of survey items for each topic are provided in the reference associated with each of these topics:

1. days lost because of poor physical health problems [34]
2. self-assessed overall health status [35]
3. health insurance status/affordability of care (Robert Wood Johnson Foundation, oral communication, in person and by telephone, 2000)
4. presence of a regular personal doctor [34]
5. utilization of health care services [36]
6. smoking behavior [34]
7. doctors advising smokers to quit [36]
8. drinking behavior [34]
9. routine retinal eye exams (diabetes sample only) [34].

We selected these variables based on the availability of external benchmarks and representation of a range of health and health care quality topics.

The psychometric reliability of the following survey scales constructed using several survey items was also assessed (these are the multi-item survey scales referred to below in the "Data Analysis" part of "Methods"). A reference for each multi-item survey scale is provided in the reference associated with each of these scales:

1. getting medical care quickly [37]
2. getting dental care quickly [37]
3. shared decision making (diabetes only) [38]
4. self-care education and support (diabetes only) [39].

### Data Analysis

We calculated response rates for the online general adult survey as the ratio of the completed sample size to the number of e-mail invitations needed to achieve this sample. The response rate for the online adult-diabetes survey was the proportion of the people completing the survey who were positively identified as having diabetes. Neither rate accounts for nonworking or dormant e-mail addresses. Telephone response rates were the ratio of completed sample size to the number of randomly-selected, working residential phone numbers that had to be called to achieve this sample size.

Survey responses for adults with diabetes were weighted using diabetes-specific age and sex distributions from the 1999 Behavioral Risk Factor Surveillance Survey [34]. General adult survey responses were weighted for age, sex, educational level, and presence of a chronic condition using distributions from the 2000 National Health Interview Survey (Robert Wood Johnson Foundation, oral communication, in person and by telephone, 2000). These distributions were used in lieu of those available from the US Bureau of the Census through the Current Population Survey (CPS) because chronic-condition status was not available from the Current Population Survey [40].

We compared weighted results from the online and telephone surveys for variables listed above to available benchmarks using either the 1999 Behavioral Risk Factor Surveillance Survey or the 1998 National Health Interview Survey. For online, telephone, and benchmarking dataset samples, we used regression analysis methods to evaluate patterns of variation across population subgroups for selected health and health care quality variables. Dependent variables for the general adult sample included health insurance status, having a regular doctor or nurse, physician counseling to quit smoking (for smokers), and poor health days in the last month. Dependent variables for the adult-diabetes sample included receipt of a routine retinal exam, use of health care services, smoking behavior, and poor health days in the last month. Independent variables were age, sex, race, education, and income, plus health insurance status and having a regular doctor or nurse, except where *health insurance* or *regular doctor or nurse* was used as a dependent variable. We compared results across samples in terms of the overall explanatory value of independent variables using the Cox and Snell generalized coefficient of determination [41]. The direction, general magnitude, and significance of the effect of each explanatory variable were also compared across samples for each dependent variable.

Each of the 4 multi-item survey scales (see *Measures,* in *Methods,* above) were evaluated for psychometric reliability using standardized estimates of Cronbach alpha [41]. SPSS version 9.0 was used to conduct data analysis [42].

## Results

### Response Rates, Response Bias, and Representativeness

Of the approximately 13400 e-mail invitations sent for the online general adult population survey, 2324 individuals responded and completed at least 80% of the survey, resulting in a 17.3% raw response rate. Based on industry norms, we estimate that at least 10% to15% of e-mail addresses are nonworking or dormant. Assuming this, the true response rate for the online general adult survey is 19% to 20%. For the general adult population telephone survey, approximately 4300 working, residential phone numbers had to be dialed to achieve the target sample size of 400. This resulted in an estimated 9.3% response rate after adjusting for nonworking and nonresidential phone numbers. Completed survey samples for the online and telephone adult-diabetes surveys were 1048 and 397 respectively.

**Table 1:** Demographic indicators for Robert Wood Johnson Foundation indicator survey: general adult population

	**2001 Online**			**2001 Telephone**			**United States Current Population Survey, %**
	**Unweighted** **(N = 2324), %**			Weighted * (N = 2315), %	**Unweighted** **(N = 400), %**	**Weighted †** **(N = 396), %**	
	**Sampled ‡**		**Actual**				
Gender §							
	Male	48.3	50.9	48.0	48.0	48.0	48.0
	Female	51.7	49.1	52.0	52.0	52.0	52.0
Age §							
	18-24	12.0	7.8	12.9	3.8	12.9	13.2
	25-44	42.6	37.0	42.1	39.4	42.1	40.9
	45-64	30.4	40.1	28.8	37.4	28.8	29.7
	65 or older	15.0	15.1	16.2	19.4	16.2	16.2
Education§							
	Less than high school	15.6	11.9	15.1	7.0	11.0	16.9
	High school/GED (General Equivalency Diploma)	33.5	24.4	33.7	23.6	37.8	32.8
	Some college	24.2	37.3	30.2	34.3	24.2	27.1
	College or more	26.7	26.4	21.0	35.1	27.0	23.2
Income§							
	Less than $15000		12.6	13.3	8.0	9.4	10.5
	$15000-$24999		17.4	18.4	14.6	18.3	12.0
	$25000-$34999		17.1	17.2	17.5	22.4	11.9
	$35000-$49999		19.1	19.5	17.5	14.5	16.5
	$50000-$74999		19.0	18.8	22.9	20.4	21.2
	$75000 or more		14.8	12.8	19.5	15.0	27.9
Race/Ethnicity §							
	White		89.6	80.0	80.0	76.0	73.3
	African-American		2.3	7.1	6.3	6.9	11.6
	Asian		1.4	1.4	2.3	2.2	3.9
	Hispanic		3.0	8.0	6.6	9.9	10.5
	Other		3.7	3.5	4.8	5.0	0.7

^*^ Weight based on 1998 NHIS (National Health Interview Survey) distribution of age, sex, education, and presence of chronic condition.

^†^ Weight based on 1998 NHIS distribution age, sex, and education.

^‡^ Distribution of original population from which the population was sampled.

^§^ Some differences between the United States Current Population Survey and both the online and telephone responding populations in this study were at the .05 level of significance.

Table 1 and Table 2 summarize the demographic characteristics of the unweighted and weighted online and telephone survey samples. Overall, respondents to the online general adult survey match the distribution of the sampled population, with some underrepresentation of individuals age 18 to 24 and overrepresentation of individuals age 45 to 64 and individuals reporting more than a high school education. Both the unweighted online and telephone general-adult completed survey samples underrepresent nonwhite individuals, those with less than a high school education, and those with incomes over $75000. Compared to the Current Population Survey, both general adult samples overrepresent individuals with a college education (or more) and incomes of $15000 to $35000. The telephone general-adult sample was more likely to underrepresent those with less than a high school education and overrepresent those with a college education. Similar results were found in both adult-diabetes samples (Table 2). However, while the telephone diabetes survey sample dramatically underrepresented individuals under age 44, and overrepresented those over age 65 and with incomes over $75000, this was not the case for the online adult-diabetes survey sample. Neither the online nor telephone methods resulted in samples properly representing racial groups with diabetes.

**Table 2:** Demographic indicators for Robert Wood Johnson Foundation indicator survey: adult-diabetes population

	**2001 Online**		**2001 Telephone**		**Behavioral Risk Factor Surveillance Survey**
	**Unweighted** **(N = 1048), %**	Weighted * (N = 1035), %	**Unweighted** **(N = 396) %**	Weighted * (N = 387), %	**Diabetes only †** **(N = 12214135), %**
Gender ‡					
	Male	45.0	46.4	38.1	46.2	48.1
	Female	55.0	53.6	61.9	53.8	51.9
Age‡					
	18-24	1.1	1.2	0.3	0.8	1.6
	25-44	19.0	16.3	2.3	14.1	13.8
	45-64	52.5	43.5	46.5	43.6	43.0
	65 or older	27.4	39.0	50.9	41.5	41.6
Education‡					
	Less than high school	7.5	7.2	12.4	11.3	26.3
	High school/GED (General Equivalency Diploma)	25.3	25.4	33.3	32.1	32.8
	Some college	41.8	41.9	28.8	29.5	21.8
	College or more	25.4	25.5	25.5	27.1	19.1
Income‡					
	Less than $15000	13.9	13.9	20.9	20.7	24.5
	$15000-$24,999	17.1	17.7	19.7	18.1	23.5
	$25000-$34,999	19.2	19.2	15.1	12.8	17.1
	$35000-$49,999	23.4	24.4	16.2	16.2	14.9
	$50000-$74,999	17.9	16.9	14.2	16.8	11.6
	$75000 or more	8.5	7.9	13.9	15.4	8.4
Race/Ethnicity‡					
	White	90.6	91.2	83.0	83.5	66.4
	African-American	3.1	2.8	9.4	9.7	14.4
	Asian	0.9	1.0	0.3	0.2	1.9
	Hispanic	2.7	2.4	3.2	2.9	14.9
	Other	2.7	2.6	4.1	3.7	2.4

^*^ Weight based on 1998 NHIS (National Health Interview Survey) distribution of age and sex of Type II diabetes population. Although the online and telephone data are both weighted to the same NHIS data, slight differences in the distributions occur because the cell for males 18-24 was 0 for the telephone sample, making it impossible to create a weight for that group.

^†^ Weighted to US population; unweighted N = 9496.

^‡^ Some differences between characteristics of the population of people with diabetes using the BRFSS and both the online and telephone responding populations in this study were observed at the .05 level of significance

### Comparison to Other National Studies

Table 3 and Table 4 compare results from both the general-adult and adult-diabetes online surveys to those obtained from the telephone surveys and benchmark data reported in other national studies. For the general adult population, the weighted online-survey results are not significantly different from those derived from the Behavioral Risk Factor Surveillance Survey and the National Health Interview Survey on 7 of the 12 health statuses, access to care, utilization of care, and clinically-appropriate health and health care quality indicators, including: (1) presence of health insurance, (2) having a regular doctor or nurse, and (3) receipt of advice to quit smoking for smokers. For the sample of persons with diabetes, results from the online survey were not significantly different from the BRFSS or NHIS benchmarks on 7 of the 13 indictors used, including (1) self assessed health status, (2) presence of health insurance, (3) having a routine checkup, (4) getting a retinal eye exam at least once in the least year, (5) receipt of advice to quit smoking for smokers, and (6) routine retinal exams for diabetics.

In the general-adult population survey, we observed higher proportions of individuals reporting 7 or more poor health days, fair or poor health status, and smoking.

In addition to comparing point estimates produced by this online survey to those produced by national benchmark datasets, we also evaluated how these datasets compare in terms of identifying variations and disparities in the health and health care quality across demographic subgroups as well as according to characteristics such as health insurance status and presence of a regular doctor. Table 5 and Table 6 present results from logistic regression analyses conducted to evaluate patterns of variation observed using data collected online versus data collected by telephone and versus telephone-based national benchmark datasets (BRFSS and NHIS).

The independent variables included in this analysis had similar explanatory power for dependent variables from the general adult survey whether data were collected using online or telephone methods. Specifically, at the low end, the demographic and health care related independent variables explained 5% to13% of the variation observed in reports of days lost because of poor health for the national dataset sample (5%), the telephone sample (13%), and the general adult online sample (9%), respectively. At the high end, these variables explained 25% to 34% of variation observed in the presence of health insurance across all datasets. For the adult-diabetes samples, on the low end, the independent variables used here accounted for less than 5% of the variation observed in rates of high utilization of health care. On the high end, these variables accounted for 11% to17% of variation observed in rates of smoking for all 3 adult-diabetes samples compared.

Along with the overall explanatory value of independent variables, we observed consistency across the general adult population datasets in terms of the approximate magnitude and significance of effect of specific independent variables. Having a regular doctor and income showed the most consistent and statistically significant effects (P < .05). Age and educational level, meanwhile, were the most consistently significant for the dependent variables evaluated for the adult-diabetes samples. No instances were found in which a variable was significant in one sample and also significant in the opposite direction in another. We did find cases of a variable being significant in one sample, but not in another. In most cases, this is attributed to chance or smaller sample size.

**Table 3:** Comparing online scores to external benchmarks and telephone data on selected health care indicators: general adult population (adjusted to the benchmark by gender and age)

**Health Care Indicators—Proportion Who:**	**Overall**			**Female**			**Male**		
	**Online** **(N = 2315)**	**Telephone** **(N = 396)**	Benchmark *	**Online**	**Telephone**	**Benchmark**	**Online**	**Telephone**	**Benchmark**
Report ≥ 7 poor health days in last 30 days	22.9	18.3‡	14.0§	26.9	20.0	15.9	18.7	16.5	11.9
Report Excellent or Very good health	45.2	56.6‡	56.1§	38.4	51.4	55.0	52.5	62.2	57.3
Have health insurance	86.3	92.7‡	85.9	87.7	94.7	86.8	84.9	90.6	85.0
Have a regular doctor or nurse:	80.9	84.5	83.3	85.2	88.9	85.3	76.2	79.7	81.3
Had routine check up (last 12 months)	63.9	68.9	70.6§	70.8	76.3	77.7	56.5	60.9	62.9
Had 1-3 doctor visits (last 12 months)†	43.3	49.7‡	44.4	43.9	48.9	43.6	42.7	50.6	45.2
Had ≥ 10 doctor visits (last 12 months)†	15.0	13.9‡	14.2	18.8	17.1	17.8	10.8	10.5	10.3
Delayed care due to cost	21.5	10.8‡	10.3§	26.0	12.5	11.8	16.7	9.0	8.6
Currently smoke	35.0	25.1‡	22.5§	34.3	21.5	20.3	35.8	29.0	24.8
Smoke and were advised to quit by doctor	52.2	50.0	55.9	57.8	56.7	61.9	46.2	42.8	50.6
Binge drink more than once per month (ie, 5 or more drinks at 1 sitting)	8.2	8.1	9.3	3.7	2.4	3.9	13.0	14.2	14.6
Average number of drinks of alcohol on typical day (drinkers only)	2.5	2.6	2.4	2.1	2.1	2.0	2.8	3.1	2.8

^*^ Weighted N for BRFSS (Behavioral Risk Factor Surveillance Survey) sample is 200000000; unweighted N for BRFSS sample is 159989.

^†^ Based on 1998 NHIS (National Health Interview Survey) data.

^‡^ Significant difference between Online and Telephone samples: < .05.

^§^ Significant difference between Online and BRFSS samples: < .05.

**Table 4:** Comparing online scores to external benchmarks and telephone data on selected health care indicators: adult-diabetes population (adjusted to the benchmark by gender and age)

**Health Care Indicators—Proportion Who:**	**Overall**			**Female**			**Male**		
	**Online** **(N = 1035)**	**Telephone** **(N = 387)**	Benchmark *	**Online**	**Telephone**	**Benchmark**	**Online**	**Telephone**	**Benchmark**
Report ≥ 7 poor health days in last 30 days	35.7	29.7‡	33.6§	37.2	32.4	38.7	32.9	26.7	28.3
Report Excellent or Very good health	18.0	25.9‡	19.1	14.5	25.6	17.3	20.3	26.4	20.8
Have health insurance	93.2	94.0‡	89.1	93.3	95.3	87.9	88.7	92.8	90.5
Have a regular doctor or nurse	96.9	95.3	91.0§	97.0	98.2	93.0	91.8	92.2	89.0
Had routine check up (last 12 months)	91.7	92.0	88.9	91.5	92.9	89.8	91.9	91.1	88.0
Had 1-3 doctor visits (last 12 months)†	24.4	24.8	22.7	22.7	27.8	20.9	23.4	21.7	24.7
Had ≥ 10 doctor visits (last 12 months)†	27.4	28.6	35.7§	31.2	29.7	38.2	22.8	27.4	32.8
Delayed care due to cost	17.6	14.0‡	12.4§	20.4	16.0	13.6	12.4	12.0	11.0
Had retinal eye exam at least once in last year	67.3	68.6	68.9	67.0	70.6	70.3	64.5	68.9	67.6
Currently smoke	19.8	16.9‡	14.6§	21.4	15.9	14.3	17.1	18.2	14.9
Smoke and were advised to quit by doctor	69.9	63.5	66.8	69.5	62.8	66.2	66.8	64.3	67.7
Binge drink more than once per month (ie, 5 or more drinks at 1 sitting)	3.3	2.0	4.2§	1.5	0	1.7	4.6	4.2	6.7
Average number of drinks of alcohol on typical day (drinkers only)	1.8	1.7	2.1	1.5	1.5	1.7	2.1	1.9	2.3

^*^ Weighted N for BRFSS (Behavioral Risk Factor Surveillance Survey) diabetes sample is 12214135; unweighted N for BRFSS diabetes sample is 9496.

^†^ Based on 1998 NHIS (National Health Interview Survey) data.

^‡^ Significant difference between Online and Telephone samples: P < .05.

^§^ Significant difference between Online and BRFSS samples: P < .05.

**Table 5:** Logistic regression results for the general adult population—comparison of odds ratios estimated using online data versus 1999 BRFSS (Behavioral Risk Factor Surveillance Survey) data and telephone supplemental data (cell values are odds ratios calculated using logistic regression analysis methods [43])

	**Has Health Insurance**			**Has Regular Doctor**			**Advised to Quit Smoking (Smokers Only)**			**7 or More Poor Health Days in Last Month**		
	**Online**	**Telephone**	**BRFSS**	**Online**	**Telephone**	**BRFSS**	**Online**	**Telephone**	**BRFSS**	**Online**	**Telephone**	**BRFSS**
Unweighted N	1992	344	4127*	1992	344	4127*	706	81	682*	1991	344	4069*
R ^2^	.34	.24	.24	.30	.26	.10	.16	.19	.10	.09	.13	.05
Predictors												
	Male	1.03	1.03	.97	.61‡	.53	.57‡	.82	.70	.62§	.81||	.84	.94
	White	1.10	1.21	1.37§	1.38	1.97	.93	1.22	.19||	1.29	.91	1.13	1.13
	Income < $25000	.37‡	.55	.19‡	.79	.42||	.88	1.15	.90	.87	2.45‡	4.19‡	2.20‡
	Education†	1.85‡	1.77	1.99‡	.79	.92	.83	.95	1.16	1.10	.68‡	.62	.64‡
	Regular doctor	12.60‡	10.30‡	4.28‡	--	--	--	5.27‡	3.16	2.31‡	2.34‡	1.90	1.94‡
	Uninsured	--	--	--	.08‡	.10‡	.24‡	.77	.31	.46‡	1.02	1.25	.81
	Age 18-44	.10‡	.36	.12‡	.18‡	.15||	.44‡	.63	.35	1.22	1.05	1.15	.87
	Age 45-64	.11‡	.28	.15‡	.41||	.33	.83	.73	.46	1.15	1.60§	1.19	1.23

^*^ BRFSS sample size is small because the question regarding having a regular doctor is asked only of a subset of subjects.

^†^ Education was grouped into high school or less, versus some college or more.

^‡^ P < .001.

^§^ P < .01.

^||^ P < .05.

**Table 6:** Logistic regression results for the adult diabetes population—comparison of odds ratios estimated using online data versus 2 external benchmarks, the 1999 BRFSS (Behavioral Risk Factor Surveillance Survey) or 1998 NHIS (National Health Interview Survey) data and telephone supplemental data (cell values are odds ratios calculated using logistic regression analysis methods [43])

	**Had Retinal Eye Exam**			**Had 10 or More Doctor Visits**			**Current Smoker**			**7 or More Poor Health Days in Last Month**		
	**Online**	**Telephone**	**BRFSS**	**Online**	**Telephone**	**NHIS**	**Online**	**Telephone**	**BRFSS**	**Online**	**Telephone**	**BRFSS**
Unweighted N	904	340	256*	903	339	529	904	340	259*	904	337	251*
R ^2^	.10	.05	.14	.04	.01	.04	.11	.11	.17	.06	.05	.05
Predictors:												
	Male	1.03	.94	.50‡	.75	1.01	.78	.81	.63	.71	.86	.85	.84
	White	1.44	1.26	.70	1.01	1.06	1.74‡	1.20	1.77	.98	1.00	1.31	1.84
	Income < $25000	.95	1.19	.94	1.81§	1.46	1.55	1.41‡	.97	2.36‡	2.00§	1.78‡	1.58
	Education†	1.22	1.52	1.52	1.01	1.11	1.61‡	.76	.57	.41	.79	.98	.68
	Regular doctor	2.11	1.14	2.18	3.13‡	1.06	1.23	1.01	1.06	1.54	.86	.15	.90
	Uninsured	.45||	.31	.20§	.86	1.32	1.18	1.45	.65	.53	.72	.21	1.02
	Age 18-44	.31§	.19‡	.57	1.02	.95	.92	5.77§	5.96‡	4.60‡	1.25	1.24	.76
	Age 45-64	.46§	.94	1.15	1.13	1.05	1.22	3.97§	3.85§	7.88§	1.82§	1.34	.91

^*^ BRFSS sample size is small because the question regarding having a regular doctor is asked only of a subset of subjects.

^†^ Education was grouped into high school or less, versus some college or more.

^‡^ P < .05.

^||^ P < .01.

^§^ P < .001.

### Scale Reliability

Cronbach alpha internal consistency scores were .72 or above for each of the 4 multi-item scales observed here (.72-.95), demonstrating their psychometric reliability when online administration is used (Table 7).

**Table 7:** Cronbach alpha reliability scores for multi-item scales using online data

**Scale**	**Cronbach Alpha**
Getting medical care quickly (CAHPS*)	
	General adult 2001	.81
	Adult diabetes 2001	.72
Getting dental care quickly (CAHPS*)	
	General adult 2001	.85
	Adult diabetes 2001	.82
Shared decision making (Diabetes PORT/ FACCT† ONE)	
	Adult diabetes 2001	.95
Self-care education and support (FACCT ONE)	
	Adult diabetes 2001	.93

^*^ CAHPS = Consumer Assessment of Health Plans

^†^ FACCT = Foundation for Accountability

## Discussion

This study found evidence that online health care surveys originally designed for mail or telephone administration maintained both psychometric reliability and concurrent validity in results across demographic and other subgroups. More specifically, estimates of access to care, utilization of care, application of clinically appropriate care, and consumer experiences of care were similar to those derived from more traditional methods of obtaining representative samples of the US population.

We were able to achieve a sample representative of the US population in terms of age, sex, and education using a readily-available, opt-in sampling frame that employs relatively low-cost recruitment methods. Basic statistical weighting methods further aligned the responding population sample on these variables. Prior information on the affiliation of individuals included in the Web panel prevented stratified sampling based on race or income. Consequently, we cannot determine whether differences between our completed survey sample and the US population in the proportion of persons representing each racial and income group are due to response biases or inadequate representation in the original sampling frame for this study. Since a great deal of concern focuses on health care for lower-income individuals, it is important keep in mind that this group was, in fact, overrepresented when compared to the US population.

Given the importance of equitably representing the range of racial and economic groups, Web-based panels used for public information about health care quality should strive to include these variables so that stratified sampling may occur and/or assessments of response bias can take place. Here, oversampling methods often used in other national studies were successful in attenuating potential biases in results caused by lower rates of representation among nonwhite racial groups.

Response rates for Internet-based, telephone, or mailed surveys must be calculated in comparable ways and take into account differences in follow-up steps with nonrespondents. In this study, while analogous administration steps were used for both the online and telephone surveys, more-robust follow-up strategy was used for the telephone survey (6 follow-up calls for telephone and no follow-up steps for online survey). In spite of this, the online response rate was higher than for telephone when comparable calculations were used. This finding is true even when nonworking and nonresidential numbers are removed from the telephone sample and similarly nonworking or dormant e-mail addresses are not removed from the online sample. Given the unique sampling and administration processes employed for both surveys, these findings may not be observed in cases where relatively-simple online methods are compared to more-complex and more-costly sampling and administration methods typical of national studies such as the National Health Interview Survey and the National Medical Expenditures Panel Survey. An important question to examine further is whether such extensive follow-up methods are required to generate public information about health care quality and whether Internet-based methods outlined here may be suitable, especially as Web access continues to expand for all population groups.

Overall, findings from this study demonstrate that many of the sampling and survey administration challenges inherent in telephone and mail modes of data collection are also present for Internet-based methods. In turn, the survey administration, statistical sampling, and weighting approaches used to ensure that data collected via telephone or through mailed surveys yield adequate and representative samples, are also required for data collected via the Internet.

Internet-based data collection is appealing in its potential for allowing information to be collected in a timely and efficient manner. These efficiencies are eroded, however, if costly strategies are required to recruit panels from which sampling may occur and/or when the survey administration process includes extensive nonresponder follow-up and tracking steps. The methods used in this study were selected to be low burden in terms of the sampling frame and administration. This was done in order to begin to explore whether the benefit of obtaining data in a timely and potentially-interactive manner using the Internet can be achieved without incurring costs that diminish the value of doing so when compared to traditional telephone methods used by most nationally-recognized studies.

As these and other issues regarding the use of the Internet to conduct health and health care quality surveys are evaluated, it is worth recalling that our comfort with telephone surveys dates only from the late 1970s, when relatively-sophisticated methodologies were established involving random-digit dialing and multiple contact strategies [24]. In fact, the rise of the telephone survey in the late 1960s and early 1970s was attended by similar methodological concerns as those now associated with Web surveys - and took a decade of research and refinement to resolve. In recent years, the growing use of unlisted numbers, cell phones, call waiting, caller identification, and answering machines have induced a steady decline in response rates and growing disparities in the populations willing to be contacted by telephone. For example, Gallagher et al found that only an elaborate and expensive combination of mail, phone, and door-to-door solicitations produced a respondent pool fully representative of the low-income community [44]. As a result, Dillman has argued that only self-administered surveys - whether made available by mail, interactive voice response, or the Internet - are likely to be successful in the coming years [19].

Results of these analyses suggest that weighted online sampling offers an imperfect but promising avenue for collecting large-scale representative survey data. Overall, conclusions about the level and variations in health care quality in the United States are similar whether based on data collected online or data collected using more elaborate and costly survey methods.

All forms of survey-based data collection involve certain sampling and mode effect biases. Tradeoffs in the biases entailed in online versus telephone based surveys need to be carefully considered by policymakers. As Internet access increases along with the propensity for individuals to resist telephone solicitations, online survey methods may increasingly represent an efficient, real-time alternative for assessing health and health care quality in the United States.

## Abbreviations

BRFSS: Behavioral Risk Factor Surveillance Survey
NHIS: National Health Interview Survey
US: United States
